# STEMSIM: a simulator of within-strain short-term evolutionary mutations for longitudinal metagenomic data

**DOI:** 10.1093/bioinformatics/btad302

**Published:** 2023-05-08

**Authors:** Boyan Zhou, Huilin Li

**Affiliations:** Division of Biostatistics, Department of Population Health, New York University School of Medicine, New York, NY 10016, USA; Division of Biostatistics, Department of Population Health, New York University School of Medicine, New York, NY 10016, USA

## Abstract

**Motivation:**

As the resolution of metagenomic analysis increases, the evolution of microbial genomes in longitudinal metagenomic data has become a research focus. Some software has been developed for the simulation of complex microbial communities at the strain level. However, the tool for simulating within-strain evolutionary signals in longitudinal samples is still lacking.

**Results:**

In this study, we introduce STEMSIM, a user-friendly command-line simulator of short-term evolutionary mutations for longitudinal metagenomic data. The input is simulated longitudinal raw sequencing reads of microbial communities or single species. The output is the modified reads with within-strain evolutionary mutations and the relevant information of these mutations. STEMSIM will be of great use for the evaluation of analytic tools that detect short-term evolutionary mutations in metagenomic data.

**Availability and implementation:**

STEMSIM and its tutorial are freely available online at https://github.com/BoyanZhou/STEMSim.

## 1 Introduction

It has been increasingly important to study within-species diversity in complex microbial communities (Van Rossum et al. 2020). To deepen our understanding of genotypic and phenotypic varieties at strain level, many tools have been developed for strain tracking and variants identification in metagenomic data (Anyansi et al. 2020). In the meantime, CAMISIM, a versatile microbial community and metagenome simulator, was designed to generate benchmark datasets (Fritz et al. 2019). It can simulate a wide range of highly diverse longitudinal metagenomic data with great flexibility and provide the gold standard for method evaluation.

As the resolution of metagenomic analysis increases, recent studies have begun to tackle the problem of tracking microbial evolution (e.g. positive and purifying selection) in longitudinal samples (Yilmaz et al. 2021; Maddamsetti and Grant 2022). Specifically, they focus on the identification of short-term mutations or monitoring the dynamic change of allele frequencies in microbial genomes. Studying the evolution of microbial genomes will facilitate to reveal the genetic mechanism of microbial response to environment factors, such as antibiotic persistence (Huo et al. 2022). While metagenomics simulator (e.g. CAMISIM) has been widely used in benchmarking studies for taxonomic classifications, the tool for simulating within-strain evolutionary signals in metagenomic data is still lacking. Here, we introduce STEMSIM (short-term evolutionary mutations simulator), which can generate mutations including single nucleotide variant (SNV), insertion, and deletion (InDel) with various frequency distributions within strains in raw metagenomic sequencing data under a specified nucleotide substitution model. Thus, it provides a command-line software to generate simulated dataset to assess methods for detecting short-term evolutionary mutations in metagenomic data.

## 2 Methods and software implementation

For the existing sequencing reads simulators (Huang et al. 2012; Fritz et al. 2019), the basic idea of simulating metagenomic data is to generate raw sequencing reads from a set of microbial genomes based on the given scenarios. For example, users need to provide the genomes of multiple strains to generate within-species diversity, or provide strain genomes with the specified genetic distances to simulate long-term mutations which are fixed in the evolutionary history between strains. CAMISIM can easily perform such tasks and automatically produce varying evolutionary genome divergence. However, in this study, we focus on the within-strain mutations and de novo mutations in longitudinal metagenomes, which we refer as potential short-term evolutionary mutations (SNVs and InDels). For instance, the mutations under positive selection may have increasing allele frequencies (e.g. 0.2, 0.5, 0.8, 1) in the longitudinal samples, while the mutations under purifying selection may have decreasing allele frequencies (e.g. 0.8, 0.5, 0.3, 0) (Biswas and Akey 2006). In contrast to the fixed mutations, those distinct allele frequency trajectories of short-term evolutionary mutations can hardly be incorporated by the existing simulators including CAMISIM, because these simulators usually can only specify one sequencing depth for one genome, i.e. the same allele frequency trajectory for all mutations on the same genome.

One possible solution is to divide the complete genome of a target strain into hundreds or thousands of fragments according to the required mutation number. Then, one can simulate different patterns of allele frequency by setting the depths of fragments to different values. However, this approach breaks the continuity of genome and is too tedious to be incorporated in the current pipelines. To deal with this problem, we develop STEMSIM which can generate potential short-term mutations in the simulated longitudinal metagenomic data. STEMSIM mainly consists of three steps: (i) align the simulated reads to their original reference genomes, (ii) determine which reads and which bases on the reads should be mutated according to the given parameters in the alignment files (sam/bam), and (iii) record the detailed information of mutations and modify the simulated reads accordingly.

**Figure 1.**
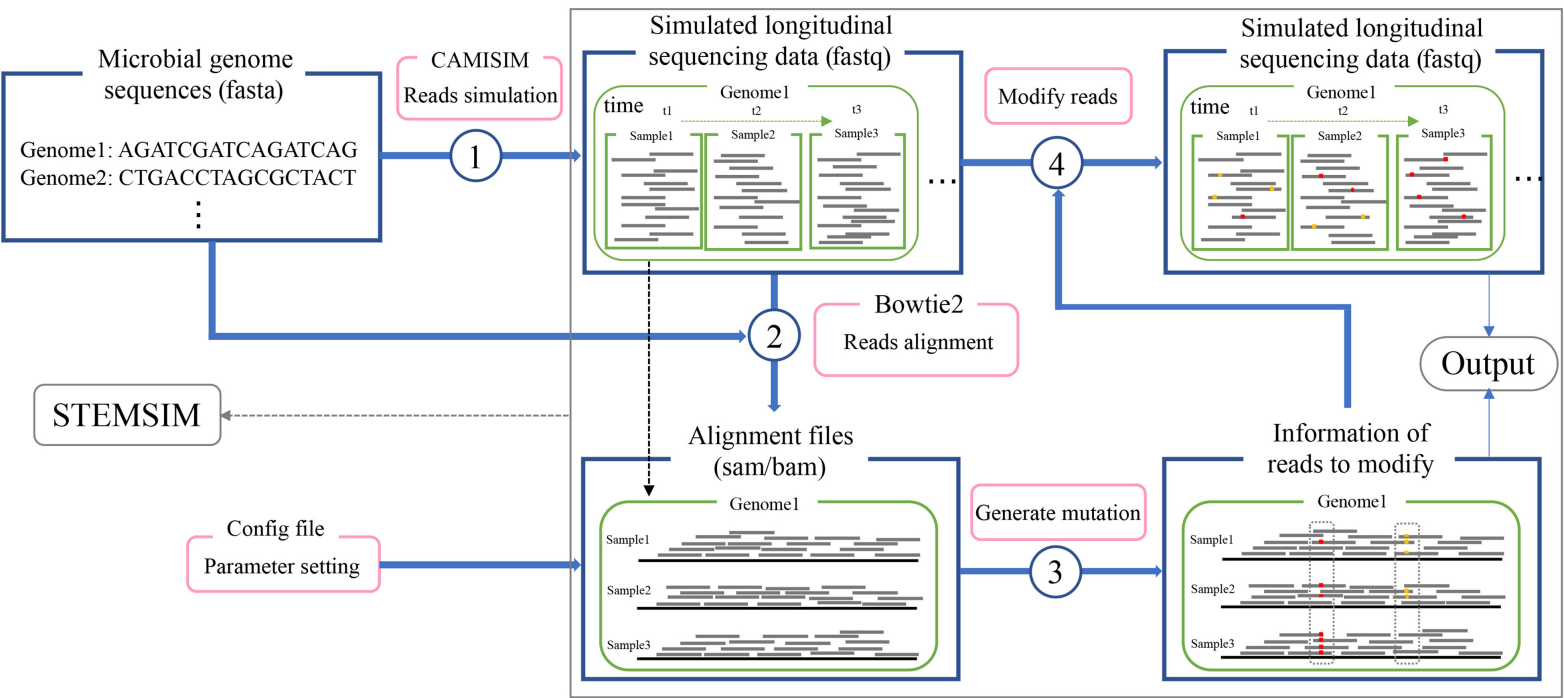
The workflow of STEMSIM. Step 1: Simulate longitudinal samples (fastq) of microbial communities by CAMISIM (recommended) according to the given reference genomes (fasta). Step 2: Align the raw sequencing reads to the genomes where they were simulated from by Bowtie2 to obtain the alignment files (sam/bam). Step 3: Generate and record the locations of the mutations on reads according to the parameter setting. Step 4: Modify the original simulated reads according to the read IDs and mutation positions recorded in Step 3. The final output are the simulated reads (fastq) with mutations and the information of mutations (e.g. position in the genome and base type).

The workflow of STEMSIM is illustrated in Fig. 1. STEMSIM is designed with flexible compatibility. It can handle the raw reads (fastq or unaligned bam) generated by other software (Huang et al. 2012) as long as their original reference genomes are provided. Since CAMISIM is a well-established and widely used pipeline which can meet the needs of most situations, in the default setting, STEMSIM directly takes the output of CAMISIM as input data. Next, the raw sequencing reads are mapped to the original reference genomes to obtain the alignment files (sam/bam) by Bowtie2 (Langmead and Salzberg 2012). Then, the details of mutations are generated according to the specified parameters, such as the number of nucleotide substitutions, and the distribution and trajectory of allele frequency. Particularly, for generating SNVs, the nucleotide substitution model can be chosen from some standard models of nucleotide evolution, including JC69, K80, HKY85, TN93, and REV (Whelan et al. 2001). The distribution of allele frequency can be manually inputted or generated from beta distribution. Given the allele frequency of each mutation in the longitudinal samples, the reads carrying mutations are randomly selected by Bernoulli process. All mutations are randomly distributed across genome unless specified by user. In this step, the positions of mutation in the genomes and the corresponding reads covering these positions are extracted from the alignment files and recorded. In the last step, the corresponding reads in the original simulated data are modified to carry mutations and other reads remain unchanged. The output of STEMSIM includes raw metagenomics data with mutations, as well as the detailed information of these mutations in the Variant Call Format (VCF).

## 3 Results and discussion

STEMSIM is a command-line software written in Python3 and is designed to work on Linux operating systems. With a 2.40 GHz CPU, STEMSIM can process one million reads in three minutes on a server. It can be used in combination with CAMISIM to generate within-strain mutations in longitudinal microbial communities or works solely on simulated or real raw metagenomic data. The parameters of the generated mutations are highly customizable and can be easily changed via the configure file (a detailed tutorial is available on the STEMSIM GitHub Wiki). By providing a user-friendly mutations simulator in metagenomic data, we believe that STEMSIM will be widely used and facilitate the evaluation of analytic tools at the strain level.
